# Cryptococcal Infection Across Disease Stages: Host–Pathogen Interactions, Tissue Niches, and Translational Priorities

**DOI:** 10.3390/pathogens15090902

**Published:** 2026-08-27

**Authors:** Feihong Lai, Xiaozhuo Dong, Enqi Zhao, Yangyu Zhou, Ziqi Zhao, Yiran Liang, Yuhan Zhou, Xinli Xiang, Linju Xia, Qiqi Wang, Qin Zhang, Ke Wang, Xinying Xue

**Affiliations:** 1 Department of Pulmonary and Critical Care Medicine, The First Affiliated Hospital of Guangxi Medical University, Nanning 530021, China; 2 Department of Respiratory and Critical Care, Shandong Second Medical University, Weifang 261053, China; 3 Department of Respiratory and Critical Care, Xuanwu Hospital of Capital Medical University, National Clinical Research Center for Geriatric Diseases, Beijing 100053, China; 4 Department of Respiratory and Critical Care, Emergency and Critical Care Medical Center, Beijing Shijitan Hospital, Capital Medical University, Beijing 100038, China

**Keywords:** cryptococcosis, *Cryptococcus neoformans*, host–pathogen interactions, fungal pathogenesis, disease progression, blood–brain barrier, cryptococcal meningitis

## Abstract

Cryptococcosis often begins in the lungs and may later reach the central nervous system, where it can lead to severe or fatal disease. The course of infection is shaped by changing interactions between host cells, fungal adaptive states, and local tissue environments. Many virulence factors and immune responses have been studied in detail, but the evidence remains divided across organs, cell types, and experimental systems. As a result, pulmonary infection is better understood than later stages, including dissemination, crossing of the blood–brain barrier, CNS involvement, and long-term persistence. In this review, we use a stage-resolved phenomic framework to connect host cell states, fungal adaptive phenotypes, and tissue niches throughout the course of infection. Evidence from multi-omics studies, spatial analyses, cellular experiments, animal models, and clinical cohorts is brought together to show how phenotypic features differ between stages and how uneven the current evidence base remains. The strongest evidence remains concentrated in pulmonary infection, whereas dissemination, crossing of the blood–brain barrier, and CNS disease are supported by more model-dependent data. Evidence for persistence and reactivation is the most limited. By organizing these findings into a disease-course map, this review outlines testable questions for biomarker discovery, host-directed therapy, and risk stratification in cryptococcal infection.

## 1. Introduction

Cryptococcosis is caused primarily by members of the *Cryptococcus neoformans* species complex and the *Cryptococcus gattii* species complex. Infection usually starts in the lungs, but in some patients the fungus enters the bloodstream and spreads to other organs, especially the central nervous system, where it can cause cryptococcal meningitis [1,2,3,4]. Disease progression reflects the changing relationship between host immunity, fungal adaptive phenotypes, and local tissue environments across different stages of infection [5,6,7,8]. Both species complexes are genetically heterogeneous, and genetic lineages within these complexes differ in epidemiology, host associations, and pathogenicity-related phenotypes [3,7,9]. These biological and taxonomic differences highlight the potential value of species- or genotype-resolved identification, although its independent prognostic significance remains to be established.

Many studies have examined cryptococcal virulence, host immunity, and organ-specific disease, but these findings are often discussed separately according to tissue site or experimental model [5,6,8,10]. This makes it difficult to view cryptococcosis as a connected disease course, from early lung infection to dissemination, CNS invasion, and later persistence [11,12,13,14].

Advances in single-cell transcriptomics, spatial omics, high-content phenotyping, and experimental infection models now permit higher-resolution analysis of host–pathogen interactions [15,16,17,18]. However, these approaches are still applied mainly within individual compartments or disease stages, and direct longitudinal links across the infection trajectory remain uncommon [14,19,20,21].

To address this limitation, we use stage-resolved phenomics as an evidence-organizing framework that integrates host–cell states, pathogen adaptive phenotypes, tissue niches, and evidence maturity across pulmonary infection, hematogenous dissemination, BBB crossing, CNS infection, and persistence/reactivation. Rather than proposing a new omics technology or a quantitatively complete phenome, this framework places fragmented findings within a continuous disease trajectory, distinguishes well-supported mechanisms from model-dependent hypotheses, and identifies cross-stage gaps that can be reformulated as testable translational questions.

## 2. Review Methods and Conceptual Framework

### 2.1. Literature Search and Screening Strategy

This study primarily searched PubMed and Web of Science, with Google Scholar as a supplementary source, and set the search cut-off date to May 2026. Search terms included *Cryptococcus neoformans*, *Cryptococcus gattii*, cryptococcosis, pulmonary cryptococcosis, hematogenous dissemination, blood–brain barrier, cryptococcal meningitis, single-cell RNA sequencing, spatial transcriptomics, immune phenotyping, proteomics, microglia, dormancy, viable but non-culturable state, and trained immunity. Search terms were combined according to the disease stage and research question being addressed.

The literature included in the discussion comprises basic experimental studies, animal infection models, in vitro blood–brain barrier models, organoid studies, clinical cohort studies, single-cell or spatial omics studies, and key reviews closely related to the mechanisms of *Cryptococcus* infection. Literature screening prioritized studies that provided clear information on disease progression stages and addressed host cell states, pathogen adaptive phenotypes, tissue localization, functional validation, or clinical outcomes. Studies lacking clear stage-specific or phenotypic information were cited only as supplementary references in the disease background or classical mechanisms sections.

This article is a narrative review aimed at integrating evidence and constructing a conceptual framework; it is not a systematic review or meta-analysis in the strict sense. Therefore, the literature review focuses on the stage-specific phenotypic information provided by the studies and the nature of the evidence, rather than on counting the number of studies or pooling effect sizes.

### 2.2. Types of Evidence and Standards for Assessing Strength

We considered four types of evidence: high-resolution human data, functional experimental evidence, evidence inferred from models, and clinical correlative evidence. These groups were used to describe what each study could contribute, not to rank one type of evidence above another. Human single-cell, spatial, or longitudinal studies were useful for linking findings to disease in patients; intervention, knockout, or pathway-blocking experiments helped support causal interpretation; animal, cellular, and organoid models were used to examine mechanisms in more detail; and clinical studies helped relate phenotypes to disease severity, treatment response, or outcome.

Evidence maturity was judged across four dimensions: sample relevance, phenotypic resolution, temporal continuity, and functional validation. A stage was considered relatively strong when several independent studies provided cell-resolved or functional evidence, including at least some human-relevant data; intermediate when coherent mechanisms were supported mainly by animal or in vitro models, organoids, or cross-sectional clinical associations; and limited when evidence was sparse, indirect, or lacked longitudinal and functional corroboration [15,17,21,22].

These labels are qualitative and comparative within this review. Human observational data were not automatically ranked above mechanistically decisive experimental studies; confidence instead reflected convergence across complementary evidence sources. The resulting assessment reflects relative evidence maturity rather than a numerical score or meta-analytic estimate.

### 2.3. Conceptual Framework and Schematic Logic of Stage-Based Classification

We organised cryptococcal infection into five connected stages: pulmonary infection, hematogenous dissemination, BBB crossing, CNS infection, and persistence/reactivation. These stages provide a conceptual disease-course sequence for organising the evidence rather than implying that every infection necessarily progresses through all five stages. Across this sequence, evidence is organised according to three biological dimensions: host–cell states, pathogen adaptive phenotypes, and tissue or molecular niches.

**Figure 1:**
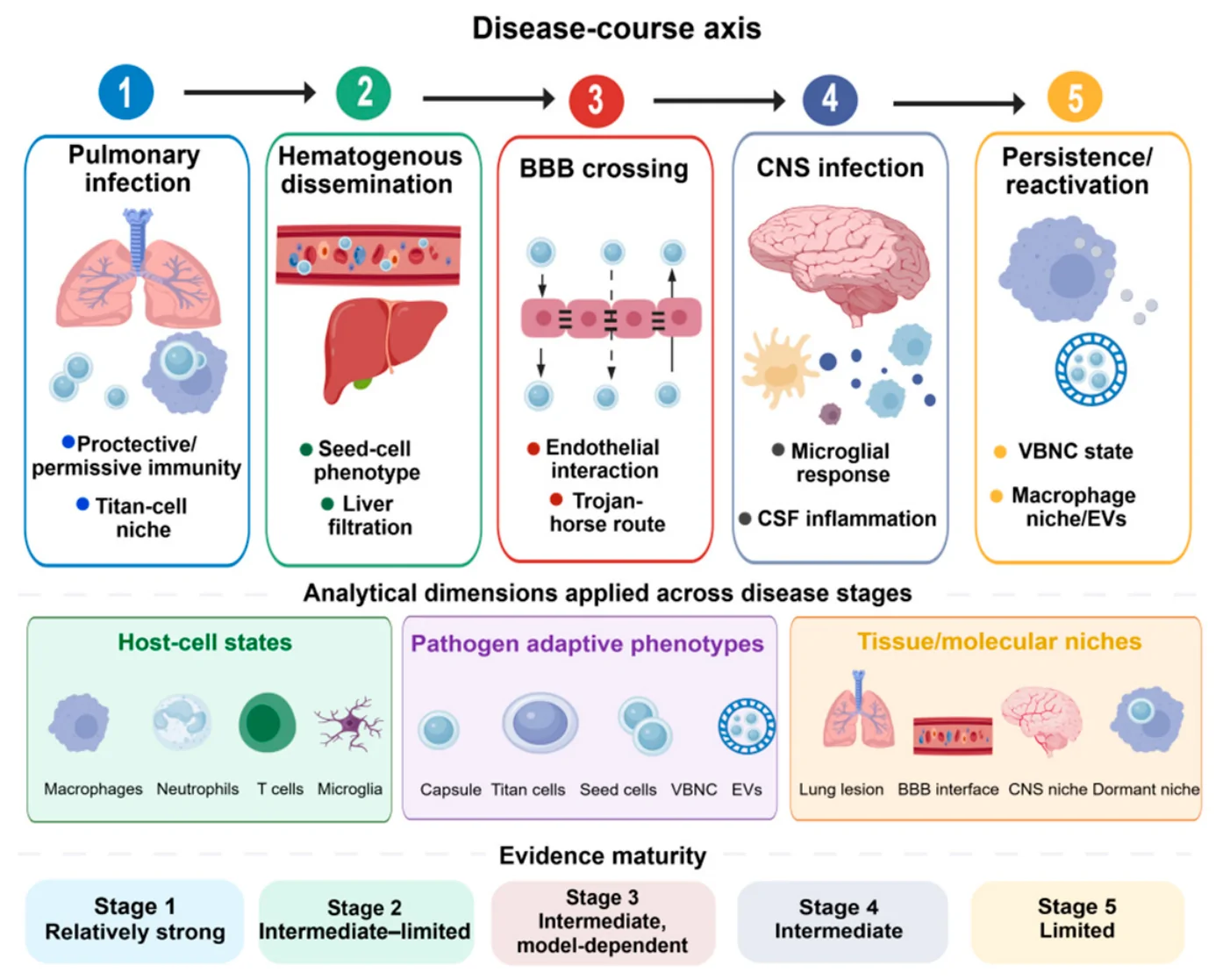
Stage-resolved phenomic framework of cryptococcal infection. The disease course is organised into five connected stages—pulmonary infection, hematogenous dissemination, BBB crossing, CNS infection, and persistence/reactivation. Across these stages, evidence is organised according to three biological dimensions—host–cell states, pathogen adaptive phenotypes, and tissue or molecular niches—together with a comparative assessment of evidence maturity. Evidence maturity is shown separately for each stage and corresponds to the qualitative assessment described in Section 2.2. The disease-course axis is used as a conceptual framework for organising evidence and does not imply that every infection necessarily progresses through all five stages. Created with BioGDP.com [23].

Figure 1 illustrates how the disease-course stages are linked to these three biological dimensions. Evidence maturity is assessed separately for each stage using the criteria described in Section 2.2.

## 3. Pulmonary Infection Stage

The lungs are the primary site of *Cryptococcus* infection and currently represent the stage with the most extensive multidimensional phenotypic evidence [11,15,20,24]. After entering the host via the respiratory tract, *Cryptococcus* first interacts with alveolar epithelial cells, alveolar macrophages, interstitial macrophages, neutrophils, dendritic cells, and local lymphocytes [11,15,25,26]. The immune response in the lung area is likely determined by whether the microorganism can be engulfed and, more significantly, whether the host can form an effective protective immune system; at the same time, some microorganisms may also be able to adapt and survive in the local environment [11,15,20,24]. Pulmonary infection may be cleared, enter a latent or chronic state, or lay the groundwork for subsequent hematogenous dissemination [11,15,26,27]. Therefore, the pulmonary stage can be regarded as a crucial bifurcation point in the development of *Cryptococcus* infection [11,12,20].

### 3.1. Pulmonary Macrophage Heterogeneity and Functional States

Traditional views typically regard pulmonary macrophages as defensive cells in the early stages of *Cryptococcus* infection, with their primary functions believed to be phagocytosis and killing of the pathogen [11,15,20,24]. However, recent immune-phenotyping and single-cell studies have shown that pulmonary macrophages are not functionally homogeneous but comprise multiple subsets with distinct inflammatory tendencies, metabolic states, antigen-presenting capabilities, and immunoregulatory functions [11,15,24,28]. There are significant differences among different macrophage populations in their ability to recognise, phagocytose, and intracellularly kill *Cryptococcus*, as well as to amplify inflammatory signals; this heterogeneity influences the course of pulmonary infection [25,29].

From the perspective of host protection, a Th1-biased immune environment, effective antigen presentation, and responses associated with classically activated macrophages generally favor the control of *Cryptococcus* [11,27,30,31]. Conversely, a Type II-biased immune response, alternative macrophage activation, excessive immunoregulation, or local tissue-repair-like reactions may create a permissive microenvironment that favors the intracellular survival and persistent proliferation of *Cryptococcus* in the lungs [11,27,32,33]. Therefore, the core of a pulmonary infection is not whether immune cells are activated, but rather the particular state of activation into which they are transformed [15,24,27]. In certain cases, a strong or dysregulated pulmonary immune response does not equate to effective clearance; rather, it may promote tissue damage or the persistence of the fungus [27,34]. Given their distinct anatomical niches and functional roles, alveolar and interstitial macrophages are considered separately below.

#### 3.1.1. Alveolar Macrophages: Protective Defence and Permissive Niche

Alveolar macrophages are among the first host cells to come into contact with inhaled *Cryptococcus* and play a central role in the early stages of infection [11,15,24]. On the one hand, alveolar macrophages can recognise pathogens via pattern recognition receptors, phagocytose *Cryptococcus*, and initiate an inflammatory response, while also participating in antigen presentation, chemokine secretion, and subsequent activation of the adaptive immune response [11,15,24]. At the same time, *Cryptococcus* can also exploit macrophages as an intracellular niche, maintaining infection by adapting to the phagosome environment, resisting intracellular killing, and undergoing nonlytic exocytosis [29,35,36,37].

This dual role indicates that alveolar macrophages cannot be simply defined as protective cells; their functional outcomes depend on the local immune context and cellular state [11,15,24]. When the pro-inflammatory signals IFN-γ and TNF-α are dominant, macrophages are in a classically activated state and enhance phagocytosis and fungicidal activity, as well as antigen presentation and pro-inflammatory effects [15,24,30,38]. Conversely, when type II immune signals such as IL-4 and IL-13 predominate, alveolar macrophages may shift toward an alternative activation pathway, exhibiting tissue repair and immune regulation, but with weaker antifungal activity [27,32,33]. For *Cryptococcus*, the latter provides a more favorable environment for intracellular survival and proliferation [27,29,35].

Therefore, the central focus of alveolar macrophage research should shift from determining whether phagocytosis occurs to analysing the cellular state following phagocytosis [11,15,24,27]. Paired spatial profiling of macrophage states, fungal burden, and local cytokine signals within the same pulmonary lesion is needed to distinguish protective macrophage programs from persistence-permissive niches [15,17,18,28].

#### 3.1.2. Interstitial Macrophages and Pulmonary Microenvironment Remodeling

Unlike alveolar macrophages, which are directly exposed to the airspace environment, interstitial macrophages are located within the pulmonary stroma. They are better understood as regulators of local inflammation spread, immune cell recruitment, and tissue microenvironment remodeling [11,15,20,24]. During *Cryptococcus* infection, interstitial macrophages may contribute to cytokine production, antigen presentation, amplification of local inflammatory signals, and tissue repair-like responses [11,15,20,24]. Changes in their numbers and functional shifts not only affect pathogen clearance but also influence the entry of other immune cells into lung tissue [11,15,27,32].

At present, it has been found that interstitial macrophages are also highly state-plastic [11,15,20,24]. In a protective immune context, they can help amplify the antifungal response. In contrast, in a permissive or chronic environment, they may also participate in the formation of a local niche conducive to the long-term persistence of *Cryptococcus* through immunoregulation, tissue repair, and alternative activation pathways [20,27,32,33]. Compared with alveolar macrophages, interstitial macrophages may more directly influence lesion structure, cellular spatial organisation, and the persistence of local inflammation [11,15,20,24].

Therefore, pulmonary *Cryptococcus* infection cannot be understood solely in terms of phagocytic events within the airways; attention should also be paid to the remodeling of the lung microenvironment [11,20,25,27]. Particularly during latent infection and the formation of granulomatous structures, interactions among interstitial macrophages, monocyte-derived macrophages, T cells, and local stromal cells may determine whether *Cryptococcus* remains confined to the local site or spreads further [20].

### 3.2. Neutrophils as Context-Dependent Antifungal Effector Cells

Neutrophils are important effector cells in pulmonary *Cryptococcus* infections and participate in antifungal defence through phagocytosis, oxidative killing, and the release of inflammatory mediators [11,28,34]. Neutrophils can directly inhibit the proliferation of *Cryptococcus* and alter the surrounding immune environment by secreting cytokines and chemokines in *Cryptococcus* infection [28,34,39]. However, the role of neutrophils is not solely protective [34,39]. Various studies suggest that neutrophil function in *Cryptococcus* infections is highly context-dependent; specifically, under certain conditions, they help control the pathogen, whereas under others, an excessive neutrophil response may exacerbate inflammatory damage and even lead to immunoregulatory or immunosuppressive states [28,34,39].

Studies at the single-cell level further indicate distinct transcriptional states and functional subpopulations within neutrophils [11,15,20,24]. Some subpopulations are biased toward direct antifungal activity and oxidative stress responses, while others may be involved in cytokine production, regulation of inflammation, or tissue damage [28]. This implies that simply comparing neutrophil counts is insufficient to explain infection outcomes; it is more important to determine their functional state and how this state interacts with the phenotypes of local macrophages, T cells, and the pathogen [11,15,20,24].

### 3.3. Pathogen Adaptive Phenotypes in Pulmonary Lesions

The pulmonary stage is where the host immune response first takes shape and where *Cryptococcus* begins to adapt to the host environment [40,41,42,43]. In the lungs, the fungus encounters surfactant, phagocytic pressure, limited nutrients, oxidative stress, and local cytokine signals [11,26,28,40]. Survival in this setting depends on changes in fungal phenotype, including capsule enlargement, cell wall remodeling, melanisation, extracellular vesicle release, and shifts in cell morphology [40,41,42,43]. These changes reflect ongoing pressure from the lung microenvironment rather than separate, unrelated events [44,45,46]. Capsule enlargement can reduce phagocytosis and alter immune recognition, melanin helps the fungus resist oxidative stress, and extracellular vesicles may carry virulence-related molecules that influence local immune responses within the lesion [45,46,47,48].

Titan cells are a prominent fungal morphotype seen during pulmonary infection [49,50,51,52]. Their large size makes them less easily phagocytosed, and their presence can change local immune pressure and the structure of the fungal population [49,50,51,52]. Titan cell formation is not simply an increase in cell size; it also involves alterations in the cell wall, capsule, ploidy, and the way the fungus interacts with host cells [48,50,51,52]. Smaller daughter cells, or other fungal cells with stronger dissemination potential, may then have an advantage during later spread through the bloodstream [12,49,50]. This means that a single pulmonary lesion may contain several fungal phenotypes, each contributing differently to persistence or dissemination [12,14,48].

Therefore, phenotypic profiling during the pulmonary phase of infection should not only document the composition of host immune cells but also simultaneously record the state of *Cryptococcus* itself [12,14,17,19]. Future studies that can simultaneously analyse the host’s single-cell state, local spatial structure, and the pathogen’s morphological and metabolic phenotypes within the same lesion will help determine which local microenvironments promote fungal clearance and which niches contribute to chronicity and dissemination [12,14,17,18].

### 3.4. An Integrated Understanding of the Pulmonary Phase

Overall, the pulmonary phase is a critical branching point where the host cell state, the pathogen’s adaptive phenotype, and the tissue niche collectively shape the outcome of infection [11,12,20,27]. The key unresolved question is how pulmonary immune niches shape fungal phenotypes that either remain locally contained or acquire dissemination potential [12,17,19,20].

## 4. Hematogenous Dissemination and Crossing of the Blood–Brain Barrier

Entry of *Cryptococcus* into the bloodstream and subsequent CNS invasion marks the transition from localised pulmonary infection to disseminated disease [12,53]. Compared with the pulmonary stage, high-resolution evidence for hematogenous dissemination and BBB crossing remains limited. Current understanding derives mainly from dissemination-prone fungal phenotypes, animal models, endothelial and organoid systems, and peripheral-blood transcriptomic studies [12,22,53,54]. This stage therefore contains numerous mechanistic clues but few continuous human phenotypic maps [12,22,55].

### 4.1. Pathogen Dissemination Phenotypes: From Local Adaptation to Extrapulmonary Invasion

Cryptococcal dissemination is not merely a passive consequence of increasing pulmonary fungal burden [12]. Dissemination-prone morphotypes, including seed cells, can emerge within fungal populations and display enhanced extrapulmonary organ entry [12]. Their smaller size and invasive behaviour indicate that phenotypic heterogeneity can generate subpopulations with selective advantages during organ dissemination [12,14,49,50].

Capsule remodelling, changes in cell wall, extracellular vesicle release, melanisation and stress tolerance can also affect the survival of seed cells in the bloodstream [40,41,42,43]. The circulatory environment exposes *Cryptococcus* to complement, antibodies, phagocytes, shear stress, and nutrient limitation [14,53]. Dissemination should therefore be viewed as a dynamic selection process between fungal adaptive phenotypes and host circulatory defences [12,14,53].

### 4.2. Circulatory Filtration and the Hepatic Macrophage Barrier

The bloodstream is not merely a passive transport route through which *Cryptococcus* reaches the central nervous system [12,22,53]. After entering the circulation, fungal cells encounter the mononuclear phagocyte system and tissue-resident macrophages. Hepatic Kupffer cells capture circulating *Cryptococcus* within the liver sinusoids and restrict fungal access to organs such as the brain through complement-dependent recognition and phagocytosis [53].

These findings indicate that disseminated cryptococcosis depends not only on fungal entry into the bloodstream but also on the efficiency of host circulatory filtration [12,22,53]. Impaired hepatic macrophage function or fungal phenotypes that evade macrophage capture may therefore increase the likelihood of extrapulmonary dissemination. Future studies should determine how immune status, underlying disease, fungal phenotype, and antifungal treatment alter Kupffer-cell filtration and subsequent CNS invasion [12,53,54,56].

### 4.3. Endothelial Mechanisms of Blood–Brain Barrier Crossing

Current evidence supports several non-mutually exclusive routes of BBB crossing. Free fungal cells may undergo transcellular endothelial passage through receptor-mediated internalisation and transcytosis involving CD44- and EphA2-associated pathways [57,58,59]. Altered barrier permeability and urokinase–plasmin-related mechanisms may facilitate paracellular or barrier-disruptive entry [60]. Urease-dependent microvascular sequestration may prolong fungal retention in cerebral vessels and increase the probability of subsequent CNS invasion [61,62]. Macropinocytosis may provide an additional endothelial transport programme, but this mechanism remains supported mainly by experimental models [22].

Together, the above studies suggest that BBB crossing is an active host–pathogen interaction and not just a failure of the physical barrier. However, direct human evidence remains sparse. The transcriptional states of infected brain endothelial cells, the participation of local immune cells, and the temporal relationship between barrier dysfunction and intracerebral inflammation have not yet been resolved by longitudinal or spatially explicit studies [12,22,53].

### 4.4. Trojan-Horse Trafficking and the Relative Contributions of Entry Routes

Cryptococcus lives inside monocytes or macrophages in the Trojan-horse model and crosses the blood–brain barrier (BBB) by moving along host cells [63,64,65]. This pathway can shield the fungus from soluble immune factors and reframes phagocytes as both antifungal effectors and potential dissemination vehicles. It should be regarded as complementary to, rather than mutually exclusive with, free-fungal transcellular and paracellular routes [22,61,63,65].

A major limitation is that most studies rely on terminal brain fungal burden, reductionist crossing assays, or short-term microscopy. Dynamic systems capable of simultaneously tracking fungal cells, endothelial states, and migratory phagocytes are still lacking [12,22,53]. In vivo imaging, lineage tracing, single-cell multi-omics, and spatial proteomics could place free-fungal and cell-mediated crossing within the same experimental framework and estimate their relative contributions.

BBB traversal should therefore be interpreted as a context-dependent combination of endothelial internalisation, barrier permeability changes, microvascular sequestration, and phagocyte-mediated transport. Their relative importance is likely to vary with fungal phenotype, host immune background, and disease stage, and remains to be established in human-relevant dynamic models [22,57,61,63].

## 5. Central Nervous System Immunophenotypes, Clinical Findings, and Inflammatory Amplification in Cryptococcal Meningitis

Once *Cryptococcus* enters the central nervous system, disease severity is determined not only by fungal burden but also by innate neuroimmune responses, peripheral immune-cell infiltration, the cerebrospinal-fluid inflammatory environment, and neural-tissue injury [13,16,21,55]. Cryptococcal meningitis is a major cause of death and neurological sequelae in cryptococcosis [1,2,66]. However, our current understanding of its high-resolution central nervous system phenotypic profile remains incomplete [13,55,67]. Existing studies largely rely on mouse models, brain tissue pathology, immunofluorescence, cerebrospinal fluid (CSF) analysis, and limited single-cell-level analyses [13,16,21,67]. Continuous spatiotemporal evidence derived directly from human central nervous system tissue remains limited [21,55,68,69].

### 5.1. Delayed Activation and Functional Deficiency of Microglia

Microglia are the core cells of the innate immune system in the central nervous system, and theoretically, they should be responsible for the initial recognition, phagocytosis and inflammatory regulation after the entry of pathogens into the brain [13,16,21,55]. However, existing studies suggest that microglia do not play an entirely protective role in cryptococcal meningitis [13,70]. *Cryptococcus* can enter brain tissue at an early stage, but the full activation of microglia is often delayed [13,16,21,55]. Even when microglia are activated, their direct antifungal capacity may be limited; under certain conditions, they may even be associated with persistent pathogen presence and amplified inflammation [13,16,70].

This phenomenon suggests that the key paradox in central cryptococcal infection lies not in whether microglial activation occurs, but rather in whether it is timely and effective, and whether such activation exerts a genuine antifungal effect [13,16,21,55]. If microglia fail to mount an effective clearance response during the early stages of pathogen colonisation, *Cryptococcus* may gain a window of opportunity for local proliferation; by the time peripheral immune cells subsequently enter the brain in large numbers, the inflammatory response may have already shifted from a protective defence to tissue damage and neuroimmune dysregulation [13,16,21,55].

### 5.2. Peripheral T-Cell Infiltration and Proliferation of Inflammatory Microglia

In cryptococcal meningitis, peripheral immune cells, especially antigen-experienced CD4+ T cells, can enter the central nervous system and change the local inflammatory environment [13,16,21,55]. These CD4+ T cells, together with IFN-γ-related responses and inflammatory myeloid cells, appear to influence how brain immune responses develop during infection [16]. Current evidence suggests that infiltrating CD4+ T cells may push microglia toward a more inflammatory state, with stronger antigen-presentation activity and IFN-γ-responsive gene expression [13,16,21,55]. Although the above response can help limit the spread of fungus in some instances, severe inflammation does not necessarily promote the clearing of fungus; instead, it may cause neuroinflammation and other damage to the tissue [16].

Based on the above, it can be seen that the immune response in the central nervous system at different times and with varying degrees of inflammation is not uniformly beneficial or detrimental [13,16,21,55]. A controlled CD4+ T-cell response together with appropriate macrophage activation may help restrict fungal growth, while delayed or excessive inflammation can worsen meningeal injury [13,16,21,55]. For CNS studies, the main challenge is to identify which neuroimmune responses contribute to fungal control and which mainly cause tissue damage [13,16,21,70]. This requires studies that link T-cell responses, microglial and monocyte-derived myeloid-cell states, fungal location, and clinical outcomes within the same stage-based analysis [13,16,21,55].

### 5.3. Clinical Correlates of Cerebrospinal Fluid Immunophenotype

As human brain parenchymal tissue is rarely available in clinical practice, cerebrospinal fluid (CSF) is often employed to examine changes in the central nervous system immune system during cryptococcal meningitis [13,16,21,55]. CSF findings, such as immune-cell composition, cytokine and chemokine levels, and protein changes, can reflect the degree of CNS inflammation and may also relate to treatment response and disease course [21,68,69,71]. Increased fungal antigen load and stronger inflammatory signals in the CSF have also been correlated with greater disease severity and worse clinical outcomes [21,71,72].

CSF analysis still has clear limitations [21,55,68,69]. CSF mainly reflects immune changes in the meninges and cerebrospinal fluid space, so it cannot fully show what is happening in the brain parenchyma or at the blood–brain barrier interface [13,16,21,55]. Using CSF markers alone also makes it difficult to determine when inflammatory responses begin and which cells are driving them [13,16,21,55]. To better understand CNS inflammation, future studies need repeated CSF and blood sampling together with imaging findings and clinical follow-up [21,69,71,73].

### 5.4. Phenotypic Issues in the Context of Treatment-Related Inflammation and Immune Reconstitution

The immune system after treatment for cryptococcal meningitis shows some alterations and may be in a reduced state [1,21,66,69]. Antifungal therapy can lower fungal burden, but fungal clearance, residual antigen, CSF inflammation, and recovery of host immunity often do not occur at the same pace [21,66,69,71]. At the same time, some patients may have an increased inflammatory response or other symptoms of immune reconstitution syndrome [1,74]. Therefore, CNS injury cannot be attributed solely to the quantity of live fungi; rather, the timing and intensity of the inflammatory response, as well as the degree of regulation of this inflammation, also affect it [21,68,69,71].

From a phenomics perspective, treatment-related CNS inflammation raises a practical question: which immune changes reflect fungal control, and which mainly indicate damaging inflammation in neural tissue [13,16,21,55]. Repeated CSF measurements, including cytokines, chemokines, immune-cell subsets, and soluble proteins, may be more useful than single-time-point testing for following treatment response and estimating prognosis [21,68,69,71]. In combination with CSF data and peripheral blood transcriptomic profiles, imaging results and long-term neurological outcomes may help define more practical phenotypic groups of CNS fungal infections [13,16,21,55].

Overall, CNS-stage phenotyping should not simply divide immune responses into activation or suppression. It is more useful to ask what the inflammation is doing at a given stage: whether it helps clear the fungus, mainly causes neuroimmune tissue injury, or reflects residual antigen stimulation or immune recovery after antifungal treatment [13,16,21,55]. To make this distinction, studies need repeated sampling before treatment, during early therapy, around fungal clearance, at periods of stronger neuroinflammation, and during recovery, with these data linked to neurological outcomes [21,55,68,69].

## 6. Persistence, Reactivation, and Trained Immunity

Cryptococcal infection does not always appear as an acute disease [14,75,76]. In some cases, *Cryptococcus* can remain in the host for long periods with few or no symptoms, and may reactivate when immune control weakens [14,75,76]. Although cryptococcosis has the trait of persistence and subsequent reactivation, the specific forms of this are not well-understood, and longitudinal and functional data are still lacking [14,76]. Current work mainly focuses on fungal dormancy, the viable but non-culturable (VBNC) state, macrophage-associated niches, and the possible role of trained immunity in host–fungal interactions [75,76,77].

### 6.1. VBNC State and Pathogen Dormancy Phenotype

VBNC is a state of pathogens that are still alive but cannot be easily detected by traditional culture methods [14,75,76]. For *Cryptococcus*, factors such as nutrient deprivation, hypoxia, host immune pressure, and drug exposure may induce the pathogen to enter a reversible, dormancy-like state characterised by low metabolism and low proliferation [75]. This state helps explain why some infections remain clinically latent for extended periods and may subsequently reactivate when the host’s immunity is compromised [14,75,76].

From a phenotypic perspective, the significance of the VBNC state lies in how it changes our assessment of whether the pathogen remains present [14,75,76]. A negative conventional culture result does not necessarily indicate complete pathogen clearance; it may simply indicate that the pathogen has entered a state in which it is difficult to detect [14,75,76]. The main unresolved issue is how VBNC or dormancy-like fungal states can be detected in vivo and distinguished from true pathogen clearance [14,17,75,76].

### 6.2. Macrophage Niche and Reactivation

Macrophages play a mixed role in cryptococcal infection. Although they can help clear the fungus, they may also serve as a place where *Cryptococcus* can survive for a long time [31,35,76]. The fungus can stay inside macrophages and later leave the cell through processes such as nonlytic exocytosis without immediately killing the host cell [36,76,78]. Recent work has also linked macrophage-derived extracellular vesicles and changes in macrophage state to the reactivation of dormant *Cryptococcus* [76]. Based on the above results, the macrophage-associated niche may be involved in the reactivation, but direct proof of this link with fungal reactivation and clinical relapse is still lacking [14,75,76].

The problem that has not been solved is not that dormancy does not occur in fungi, but rather where dormant fungal states remain in the host and what causes them to reactivate pathogenic growth [14,75,76]. Current evidence still does not clearly connect these states with specific tissue niches, immune or cellular signals, treatment pressure, and later reactivation [14,17,75,76].

### 6.3. Trained Immunity: An Explanatory Framework Still Requiring Stage-Specific Validation

Trained immunity refers to the ability of innate immune cells to develop a stronger or differently directed secondary response following an initial stimulus through metabolic and epigenetic reprogramming [77,79,80,81]. This concept provides a valuable theoretical framework for understanding memory-like immune responses following *Cryptococcus* infection [77,79]. In cryptococcal infection, dendritic cells, Th1-related responses, and other protective immune states have been studied in the context of vaccine protection; however, whether these constitute trained immunity in the strict sense remains to be further validated [38,82,83].

At present, the trained immunity in cryptococcosis should be viewed as a hypothesis-generating framework for stage-specific mechanisms rather than a fully verified one [14,75,76]. Existing evidence is derived mainly from functional experiments, in vitro stimulation models, and epigenetic studies, and has not yet established how trained immunity affects pulmonary clearance, hematogenous dissemination, CNS invasion, or reactivation during natural infection [14,75,76]. Future studies should determine whether trained-immunity-like programs alter specific disease stages, such as pulmonary clearance, dissemination, CNS invasion, or reactivation, rather than treating trained immunity as a general protective concept [14,76,77,79].

### 6.4. The Relationship Between Antifungal Drug Pressure, Metabolic Remodeling, and Long-Term Persistence

The impact of antifungal drug pressure and the corresponding transcriptional regulatory network on the phenotype of the pathogen during prolonged infection should also be considered [47,84,85,86]. Pdr802-related regulation in the macrophage microenvironment may also shape *Cryptococcus*’s adaptation and virulence programs [87]. Under conditions of drug exposure, nutritional restriction, oxidative stress, and immune pressure, *Cryptococcus* may maintain low-level survival through metabolic adaptation, cell wall/capsule remodeling, and activation of stress pathways [40,41,84,85]. These changes do not necessarily manifest as classical drug resistance but may instead promote drug tolerance, relapse, or long-term persistence of residual foci [14,75,76,88]. Distinguishing drug resistance, drug tolerance, dormancy, and tissue-protected persistence will require models that link antifungal exposure to fungal metabolic state, host niche localization, and recurrence risk [14,75,76,84].

## 7. Evidence Landscape, Key Gaps, and Future Research Priorities

Evidence maturity is uneven across the infection trajectory. Pulmonary infection has the broadest combination of immunophenotypic, functional, and single-cell evidence; dissemination and BBB crossing rely more heavily on morphotype studies and experimental barrier models; CNS studies provide emerging microglial, T-cell, and CSF data but limited human spatial evidence; and persistence/reactivation remains dominated by dormancy models and mechanistic inference [11,12,13,14]. Table 1 summarises the relative maturity of this evidence, major gaps, and research priorities.

Clinical-course studies have established a substantial evidence base for the diagnosis of cryptococcal meningitis, treatment response, CSF inflammation, and immune reconstitution. Nevertheless, these data remain concentrated in CSF and peripheral blood and cannot directly resolve brain-parenchymal or BBB-interface organisation [73]. The timing of antiretroviral therapy demonstrates that treatment timing is an integral component of the disease course [89,90]. CSF inflammatory profiles, immune activation, and early mortality associations further highlight the prognostic relevance of treatment-associated immune trajectories [72,91,92].

Extracellular vesicles and nonlytic exocytosis have become important clues for understanding persistence and niche remodeling in cryptococcal infection [36,76,93,94]. Vesicles released by *Cryptococcus* can carry capsular and protein components and may influence macrophage function [93,95]. Vesicles from infected macrophages, together with vomocytosis, also show that host cells can both limit fungal growth and help move viable fungi to new sites [78,94,96,97]. Because cryptococcal extracellular vesicles can induce immune responses, they have also been considered as possible vaccine platforms, although their protective effect still needs further testing [98].

Table 1 compares the principal phenotypic evidence, relative evidence maturity, major gaps, and priority research directions across the five disease stages.

Three cross-cutting gaps emerge. First, direct links between stages are uncommon: few studies connect early pulmonary immune states with subsequent dissemination, or dissemination-prone fungal states with CNS inflammatory outcomes [12,13,17,28]. Second, longitudinal and spatially resolved human evidence is limited, particularly at the BBB and within brain tissue [18,21,22,53]. Third, host and pathogen phenotypes are rarely measured synchronously within the same niche, despite evidence that their reciprocal adaptation shapes clearance, dissemination, persistence, and reactivation [14,29,35,75].

Therefore, the next studies should be designed to address specific disease-course questions, rather than adding isolated omics layers. A convenient Design could include explicit time points, tissue or fluid source, fungal burden, host–cell state, pathogen phenotype, spatial distribution, treatment information, and clinical outcomes [17,18,21,69]. Pulmonary studies should link lesion-level immune niches with fungal-state profiling; dissemination studies should connect fungal morphotypes with mononuclear-phagocyte filtration and endothelial responses; and CNS studies should align longitudinal CSF, blood, imaging, treatment kinetics, and neurological outcomes [12,13,17,19].

The above priorities transform the evidence landscape into specific questions that can be tested by paired host–pathogen measurements and cross-validation among human samples, animal models, BBB systems, and organoids [17,19,22,53].

## 8. From a Stage-Resolved Phenotypic Atlas to Testable Translational Hypotheses

**Figure 2:**
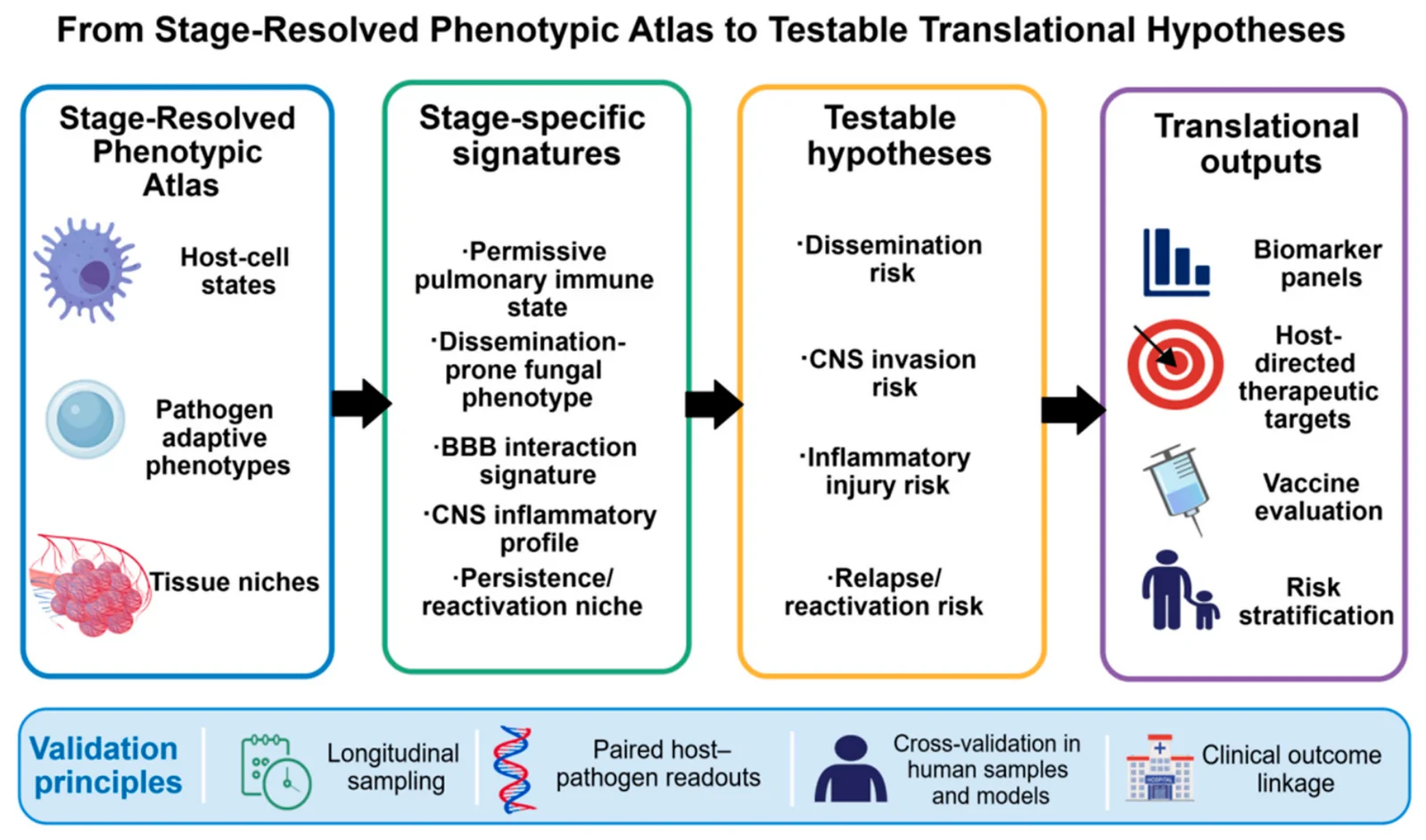
From a stage-resolved phenotypic atlas to testable translational hypotheses. Integration of host–cell states, pathogen adaptive phenotypes, and tissue niches enables the identification of stage-specific signatures and hypotheses related to dissemination, CNS invasion, inflammatory injury, and relapse/reactivation. Validation requires longitudinal sampling, paired host–pathogen measurements, cross-validation between human samples and experimental models, and linkage to clinical outcomes. Created with BioGDP.com [23].

The translational value of a stage-resolved phenotypic atlas lies in converting complex disease progression into mechanisms and risk hypotheses that can be examined in experimental models and longitudinal clinical cohorts [17,19,21,22]. Integration of host–cell states, pathogen adaptive phenotypes, and tissue niches can generate candidate signatures for dissemination, CNS invasion, inflammatory injury, and relapse or reactivation [12,13,14,17]. Figure 2 presents the progression from evidence synthesis to stage-specific signatures, testable hypotheses, and potential translational outputs.

Biomarker development and risk stratification are relatively promising directions for constructing stage-specific composite indices rather than relying on individual inflammatory or fungal markers [21,69,71,92]. Candidate profiles could integrate host–cell states, fungal burden, tissue-injury markers, and treatment kinetics. Examples requiring longitudinal validation include permissive pulmonary macrophage states and dissemination, circulating monocyte phenotypes and CNS invasion, and CSF inflammatory profiles and neurological injury or mortality [15,27,71,72].

Stage-resolved analysis can be used to distinguish protective responses that need to be strengthened from pathological responses that should be suppressed in host-directed therapy [27,31,39,99]. Pulmonary infection may benefit from effective antigen presentation and antifungal effector programs, whereas CNS disease requires a balance between fungal control and limitation of damaging inflammation [13,16,39,99]. Since the same path may have different effects at different stages, target selection should consider the stage of the disease, immune status, fungal load and tissue damage before using general immunotherapies or non-specific anti-inflammatory drugs.

For vaccine evaluation, protective pulmonary states provide a useful reference. Candidate vaccines should not be judged solely by antibody titres or a single cytokine, but also by whether they induce coordinated cellular states that restrict early pulmonary growth and dissemination [30,82,83,100]. Dendritic-cell antigen presentation, Th1/Th17-associated responses, and macrophage antifungal programs are plausible candidate readouts [30,38,82,83]. Trained-immunity-like reprogramming should be considered a separate candidate mechanism that requires direct validation in cryptococcal infection [77,79,80,81]. Several specific translational hypotheses have been developed in the stage-resolved framework for future validation in longitudinal studies and experimental models. These hypotheses are summarised in Table 2.

These translational hypotheses are proposed as a framework for research priority setting rather than as clinically validated applications. Prospective validation will need to specify the disease stage, host–pathogen measurements, spatial context, treatment information and clinically significant outcomes clearly [17,19,21,69].

## 9. Conclusions and Outlook

This paper is a narrative review to integrate concepts and build an evidence map, rather than a strict systematic review or meta-analysis. The assessment of evidence strength in this paper is primarily based on sample origin, phenotypic resolution, temporal dimension, and the extent of functional validation. Given that human spatial evidence and longitudinal data on the stages of hematogenous dissemination, blood–brain barrier crossing, central nervous system colonisation, and persistent infection are still lacking, some of the views presented should still be regarded as hypotheses derived from existing models and indirect evidence, rather than fully validated mechanisms of disease progression [12,14,22,55].

Stage-resolved phenomics helps place *Cryptococcus* infection within a disease course that changes over time and across tissue sites [12,13,14,17]. At present, the pulmonary stage has the strongest high-resolution cellular and molecular evidence, while dissemination, blood–brain barrier crossing, CNS colonisation, and persistent or latent infection are still less clearly understood [11,12,13,14]. Future work needs to follow infection across time and examine host cell states, fungal phenotypes, tissue niches, treatment exposure, and clinical outcomes together rather than as separate endpoints. This approach may clarify how an early lung infection develops into systemic spread, CNS disease, or long-term persistence, and may also provide a stronger basis for biomarker discovery, host-directed treatment, and risk assessment [17,21,22,28].

## Figures and Tables

**Table 1:** Stage-specific phenotypic evidence, evidence maturity, key gaps, and research priorities across the course of cryptococcal infection.

Disease Stage	Key Phenotypic Evidence	Evidence Strength	Major Evidence Gaps	Priority Research Directions
Pulmonary infection	Host: macrophage heterogeneity and polarisation, neutrophil subpopulations; Pathogen: capsule remodeling, titan-cell formation, stress adaptation; Niche: alveolar and interstitial pulmonary microenvironments [15,20,27,28]	Relatively strong	Lack of longitudinal and spatially continuous tracking; insufficient synchronous evidence on host phenotypes and fungal states	Time-series single-cell and spatial omics; viable-fungus localization; pulmonary niche mapping
hematogenous dissemination	Host: circulatory phagocyte responses and hepatic Kupffer-cell filtration; Pathogen: seed cells and dissemination-prone morphotypes; Niche: blood–liver–target-organ interface [12,53,54,56]	Intermediate, limited	Insufficient longitudinal human blood data; difficulty linking pulmonary states to dissemination risk	Continuous blood–liver–target-organ sampling; functional assessment of circulatory immune filtration
BBB crossing	Host: endothelial activation, receptor-mediated uptake, and monocyte/macrophage trafficking; Pathogen: urease-associated microvascular retention and endothelial-interacting phenotypes; Niche: cerebral microvascular and BBB interface [22,57,58,63]	Intermediate, model-dependent	Lack of in situ evidence on endothelial states and immune-cell involvement	Integration of human BBB models with spatial transcriptomics; dynamic tracking of endothelial states
CNS infection	Host: delayed microglial activation, CD4+ T-cell infiltration, and CSF immunophenotypes; Pathogen: local persistence under neuroimmune pressure; Niche: CSF, meninges, brain parenchyma, and perivascular regions [13,16,21,71]	Intermediate	Limited human CNS tissue evidence; CSF findings cannot be directly mapped to the spatial organization of brain parenchyma	Longitudinal CSF cohorts; single-cell neuroimmune atlases; spatial localization validation
Persistence/reactivation	Host: macrophage-associated persistent niches and trained-immunity-like responses; Pathogen: VBNC states, dormancy, drug tolerance, and nonlytic exocytosis; Niche: intracellular and tissue-protected reservoirs [36,75,76,77]	Limited	Lack of direct evidence linking persistent niches, reactivation triggers, and clinical recurrence	Pathogen metabolic-state markers; host-niche localization; long-term follow-up models

Note: Evidence maturity is a qualitative, comparative judgement based on sample relevance, phenotypic resolution, temporal continuity, functional validation, and convergence across independent studies. “Relatively strong” means that there are several complementary cellular or functional studies that include some human-relevant evidence; “intermediate” indicates that the mechanisms are consistent but mainly supported by models or cross-sectional clinical data; and “limited” means that the evidence is sparse, indirect or weakly validated. These labels are not quantitative scores. “Intermediate, limited” indicates coherent mechanistic evidence with sparse direct human support, and “intermediate, model-dependent” indicates evidence mainly obtained from experimental systems.

**Table 2:** Translational hypotheses generated by the stage-resolved phenomic framework.

Translational Question	Candidate Phenomic Signature	Required Validation	Potential Use
Which pulmonary infections are more likely to disseminate?	Permissive pulmonary macrophage states combined with dissemination-prone fungal morphotypes, such as seed-cell-like or stress-adapted phenotypes	Longitudinal lung, bronchoalveolar lavage, blood, and fungal-state profiling studies	Early dissemination-risk stratification
Which patients are at higher risk of CNS invasion?	Circulating monocyte states, endothelial activation markers, fungal burden, and BBB-interacting fungal phenotypes	Paired blood–CSF–imaging cohorts and human-relevant BBB models	Prediction of CNS involvement and early monitoring
Which CNS inflammatory responses are protective or damaging?	CSF cytokine/protein profiles combined with T-cell, microglial, or myeloid activation signatures	Longitudinal CSF studies linked to fungal clearance, neurological injury, and mortality outcomes	Guidance for adjunctive immunomodulatory strategies
Which infections may persist, relapse, or reactivate?	VBNC or dormancy-associated fungal states, macrophage-associated niche signatures, nonlytic exocytosis, and drug-tolerance phenotypes	Long-term follow-up models and recurrence-linked clinical sampling	Relapse-risk monitoring and persistence-targeted intervention
Which immune states should be prioritised in vaccine evaluation?	Coordinated antigen-presentation, Th1/Th17-associated, and macrophage antifungal programs rather than single antibody or cytokine readouts	Vaccine studies incorporating cellular phenotyping, fungal burden, dissemination endpoints, and durability of protection	Improved evaluation of protective vaccine-induced immunity

## Data Availability

No new data were created or analyzed in this study. Data sharing is not applicable to this article.
